# Suffering in silence: a qualitative study of older adults’ experiences of living with long-term musculoskeletal pain at home

**DOI:** 10.1007/s10433-020-00566-7

**Published:** 2020-04-16

**Authors:** Catharina Gillsjö, Kristina Nässén, Mia Berglund

**Affiliations:** 1 School of Health Sciences, University of Skövde, P.O. Box 408, 541 28 Skövde, Sweden; 2 College of Nursing, University of Rhode Island, Kingston, USA; 3 Academy of Care, Working Life and Social Welfare, University of Borås, Borås, Sweden

**Keywords:** Endurance of pain, Meaning in life, Loneliness, Home, Qualitative interviews, Nursing

## Abstract

Long-term musculoskeletal pain is a major, disabling, and often undertreated health problem among the increasing number of older adults worldwide. However, there is limited knowledge of community-dwelling older adults’ experiences of living with this type of pain. The aim of the study was to deepen the understanding of the phenomenon: how older adults experience living with long-term musculoskeletal pain at home. The study design was an inductive qualitative Reflective Lifeworld Research approach grounded in phenomenological epistemology. Data were obtained from 20 community-dwelling older adults, aged 72–97 years. Data were collected through open-ended interviews and analyzed to understand the meanings of the phenomenon. The essence of the phenomenon entailed suffering in silence and encompassed the following constituents: loneliness and restrictions in daily living; ways to endure and distract from pain; not being taken seriously; fear of the future; and valuing joy and meaning in life. Living with long-term musculoskeletal pain restricts access to the world and leads to a suffering in silence. Finding ways to endure and distract from pain and to focus on issues that give joy and meaning in life is predominant in efforts to balance restraints from pain in life. Suffering is reinforced by loneliness, a sense of not being taken seriously by health care providers and fear of an uncertain future. It is necessary to foster increased attentiveness and sensitivity in meeting the needs of each older adult and provide a care that alleviates suffering and preserves and promotes health and well-being.

## Introduction

Long-term musculoskeletal pain caused by musculoskeletal conditions is a global, pervasive and predominant health problem. It causes difficulties, independence, and disabilities (physical, psychological, social and existential) in daily life (Blyth et al. 2019; Cimas et al. 2018; Hoyos et al. 2018; Smith et al. 2019; Stubbs et al. 2014) with significant impact on an older adult´s sense of well-being and quality in life. Research shows that as many as 60–80% of 65-year-older adults have at least one musculoskeletal condition with potential to cause pain (Duncan et al. 2011). The pain with associated disabilities tends to increase with age (Blyth et al. 2019; Hemmingsson et al. 2018; Jackson et al. 2016). Examples of difficulties caused by pain are reduced mobility, increased risk of falls and related injuries, sleep disturbance, distress, anxiety, depression, social isolation, sense of loneliness, and loss in life (Chen et al. 2011; Crofford 2015; Hoyos et al. 2018; Nijs et al. 2020; Smith et al. 2019; Stubbs et al. 2014).

Musculoskeletal pain is a major contributor to the global burden of disability which has not been fully acknowledged and dealt with neither on an individual nor on an organizational level in the provision of health care and in healthcare policies (Blyth et al. 2019; Briggs et al. 2016, 2018; Nijs et al. 2020). It has to be recognized that pain is a subjective multidimensional experience (IASP 1986; Vallath et al. 2013) that can be described as *“whatever the experiencing person says it is, existing whenever the experiencing person says it does”* (McCaffery et al. 1968, p. 95). In addition, there is a need to take into account that healthcare problems such as pain and the situation as a whole often is accompanied with comorbidities and frailty. A holistic approach is needed in the overall management of pain to provide appropriate health care (Lohman et al. 2017; Nijs et al. 2020; Reid et al. 2015; Vadivelu et al. 2011), a care that guides and supports the older adults based on their needs (Gillsjö et al. 2013).

Despite the considerable prevalence and prevailing difficulties associated with musculoskeletal pain, researchers continue to report this type of pain and long-term pain in general, frequently being unrecognized, underestimated, underreported, and inadequately treated among older adults (AGS Panel on the Pharmacological Management of Persistent Pain in Older Persons 2009; Brown et al. 2011; Stewart et al. 2012; van der Leeuw et al. 2018), which can lead to suffering (IASP 2016). This might in part be explained by a tendency among older adults, relatives, and health care professionals to view pain as a natural part in the process of aging (Collis and Waterfield 2015). Research reveals that the undertreatment of pain among older adults is associated with the belief that pain is inevitable in late life, which adds to older adults’ reluctance to contact health care professionals for help. In addition, it seems to be a tendency among health care professionals to convey statements and behaviors that can be associated with agism (Clarke et al. 2014; Makris et al. 2015; Paskins et al. 2013). The older adults’ reticence to report pain may also be associated with existing stoic attitude (Cagle and Bunting 2017; Yong 2006). The unrecognition of pain among the older adults, health care professionals, and others might in part explains the older adult’s coaching of themselves in order to find ways to endure pain, remain as independent as possible and not burden others. Research shows that they found their own ways to deal with pain ranging from ignoring, struggling, to adjusting and resigning. However, they had a feeling of being forced into learning to live with this type of pain on their own, which needs to be acknowledged in the provision of health care (Gillsjö et al. 2012, 2013). The complexity associated in living with pain requires an integrated person-centered multimodal approach that includes services and interventions from health- and social care teams providing adequate medication, nursing interventions, psychological support, and rehabilitation. The interventions also need to include education, continuous communication, and goals defined by the patient (Briggs et al. 2018; Coghill 2010; Nijs et al. 2020). One intervention addressing these needs is the method Reflective STRENGTH-giving dialogue (Gillsjö and Berglund 2014). The method has been developed to meet individual needs using a holistic and person-centred approach to guide and support older adults’ living with long-term health problems as musculoskeletal pain.

There is an urgency and call to address the burden of musculoskeletal pain on an individual level to decrease personal suffering. Limitations in the provision of health care call for innovations and interventions to support and promote individualized and holistically tailored care. On society level, changes are needed in the health care system that accelerate health care policies and finances appropriate interventions in order to decrease the global burden of musculoskeletal pain (Blyth et al. 2019; Nijs et al. 2020). Unfortunately, research focusing on community-dwelling older adults’ experiences of living with long-term musculoskeletal pain is limited. Research is needed to understand how it is to live with this type of pain, in order to tailor individual and holistic interventions that preserve and promote health and well-being (c.f. Brown et al. 2011; Cairncross et al. 2007; Gillsjö et al. 2013; Kumar and Allcock 2008; Nijs et al. 2020; Vadivelu et al. 2011). The aim of this study is to deepen the understanding of how older adults’ experience living with long-term musculoskeletal pain at home. This study is carried out as a starting point prior to the intervention Reflective STRENGTH-giving dialogue (Gillsjö and Berglund 2014), which will be will be evaluated through follow-up interviews and questionnaires.

## Methods

This is a inductive qualitative interview study in which the Reflective Lifeworld Research (RLR) approach developed by Dahlberg et al. (2008), and grounded in phenomenological epistemology (Husserl 1989; Merleau-Ponty 2002) will be used to study the phenomenon *“Older adults’ experiences of living with long-term musculoskeletal pain at home.”* The RLR approach describes a phenomenon from a life world perspective and is commonly used in the context of caring to describe lived experience in both an abstract (essence) and concrete (constituents) level. Openness and orientation toward the phenomenon are key components in the approach, and used to guide the collection and analysis of data to describe the phenomenon as it appears. The researcher obtains openness through a conscious and deliberate “bridling” of one’s own pre-understanding and understanding of the phenomenon (Dahlberg and Dahlberg 2003; Dahlberg et al. 2008).

### Participants

Data were collected through qualitative interviews in the context of home health care in three municipalities in the Western region of Sweden. Health care professionals (nurses, physiotherapists, and occupational therapists) identified older adults that met the inclusion criteria: age 65 or above, have had long-term (persistent or regularly recurring) musculoskeletal pain for at least 6 months, and received community-based care. Additionally, they had to be able to understand and answer questions in Swedish and be willing to participate. Twenty older adults participated in the study. See Table 1 for description of study participants in regard to demographic data.

**Table 1:** Characteristics of the study participants (N = 20)

Characteristics	N	%
*Gender*		
Female	14	70
Male	6	30
*Age (year)*		
65–74	3	15
75–84	9	45
85+	8	40
*Marital status*		
Married	3	15
Widowed	13	65
Divorced	2	10
Single	2	10
*Living situation*		
Single	17	85
Cohabiting	3	15

### Data collection

The interviews were carried out as dialogues to encourage participants’ reflection and to deepen the understanding of the phenomenon (Dahlberg and Dahlberg 2003; Dahlberg et al. 2008). The interviews and reflective process were initialized by the open question, *“Would you please describe your life with long-term pain?”* The participants’ descriptions led to new thoughts and questions that were asked, and deepened with additional follow-up questions to encourage descriptions of situations in how pain influenced daily life. The interviews lasted an average of 55 min (range 23–93 min) and were audio-recorded and transcribed verbatim.

### Data analysis

The process of analysis was inductive and carried out as an open, sensitive and intensive dialogue with the text, bringing an understanding of the participants’ experiences of the phenomenon; *“Older adults’ experiences of living with long-term musculoskeletal pain at home.”* The analysis vacillated between the text as a whole and its different parts to discover patterns and qualitative meanings that deepened the understanding of the phenomenon. The continuous and conscious “bridling” of preunderstandings and understandings in the process of analysis (c.f. Dahlberg and Dahlberg 2003; Dahlberg et al. 2008) allowed various meanings of the phenomenon to emerge. Bridling encompasses adoption of an open and respectful attitude in which thoughts, feelings, opinions, and previous understandings are restrained to understand the phenomenon as it presents itself. Initially, the text was read as a whole several times for familiarization before meaning units (a word, a sentence or a longer section of text) were drawn from the text. The readings and the sense of the text as a whole helped the researchers to become involved and acquainted with the data which, in turn, facilitated the struggle to “bridle” existing preunderstandings and the understandings that evolved regarding the phenomenon. New questions were generated in the dialogue in the vacillation between the data as a whole and its different parts, which deepened the understanding of the phenomenon. The next step in the analysis was to build clusters consisting of meaningunits that were similar to each other. Initially, several clusters were created. Similarities between the clusters were identified which led to aggregation with a decrease in number. The analysis continued and the essence of the phenomenon emerged as a new whole. This essence constituted the foundation from which the five constituents were identified. The essence can be understood as the core aspects of a phenomenon on an abstract level, whereas the five constituents describe the essence of the phenomenon on a concrete level. The essence of the phenomenon is presented initially, followed by the constituents and quotes to further illuminate the findings. As suggested by Dahlberg (2006), the findings will be presented in the present tense.

## Findings

The essence of living with long-term musculoskeletal pain is characterized as suffering in silence. Living with long-term pain encompasses a sense of being forced to endure a pain that constricts daily life. The pain is reinforced by loneliness, a sense of not being taken seriously, and fear of an uncertain future. The pain forces a quiet life in which rest is used to alleviate the pain. A strive and hope for alleviation is present, although the primary orientation is to endure the inevitable pain. The act of endurance results in continuous trying out what is possible in the moment. Things that distract from pain and give joy and meaning in life are used to balance with difficulties and restrictions in living with long-term pain. The essence of the older adults’ experiences of living with long-term musculoskeletal pain is described through the following constituents:

- Loneliness and restrictions in daily living
- Ways to endure and distract from pain
- Not being taken seriously
- Fear of the future
- Valuing joy and meaning in life

### Loneliness and restrictions in daily living

Living with long-term musculoskeletal pain is associated with loneliness and restrictions in daily living. The older adults describe how the pain forces them to give up their interests, activities and social contacts. Pain in joints restricts their lives since it increases when moving: *“It aches and aches, and it hurts every step I take.”* The pain constrains life because it restricts body movements and forces them to rest in bed several times a day. Otherwise, there is a risk that the pain increases to a level that becomes overwhelming and unbearable. *“If I just move the arm a little or change position it’s like devil blasts, yes it’s like bolts of lightning”*.

Over time, restrictions due to pain in the older adults’ lives lead to increased dependence. It feels humiliating and gives a sense of losing one’s integrity and independence when not being able to perform intimate things such as personal hygiene and dressing: *“…I need two people to help me clean up and get dressed. I feel like I am a little child.”* Life with pain is also restricted through having to adjust to health care providers’ schedules. An example is not being able to go to the bathroom when needed, which is uncomfortable and results in anxiousness and frustration: *“…the first thing I say when they come, Toilet! Toilet!”* Other examples of increased dependence are related to movements and medical issues: *“I have to wait until the nurse comes for medication and help me to get up.”*

The pain and the resulting restrictions in life influence the mood: *“you become grumpy”* and *“… depressed and even irritated,”* which lead to avoidance of social interaction. Living with long-term pain contributes to a sense of loneliness, especially when spouses, relatives and friends live a social and active life, but for the older adult, the pain restricts their possibility to participate. They sometimes avoid activities and social life because they fear straining others; *“I think I’m a burden for them.”* Inactivity and loneliness contribute to a sense of insecurity in life which undergird loneliness and suffering in silence.

Restrictions caused by pain influence daily life in several ways as restricted mobility, increased dependence, lowered mood and increased loneliness, all of which contribute to social isolation.

### Ways to endure and distract from pain

Life with long-term pain requires ways of dealing with the inevitable pain: *“it never ends…there is nothing you can do about it.”* In the endurance and distraction from pain there is a tendency to resign, capitulate and incorporate the pain into the process of aging *“You become used to the pain.”* The older adults’ bodies are worn out to various extents: *“I think I take it easy. It doesn’t become better if one whines and becomes grouchy, the body is worn out.”* The older adults suffer in silence and adjust their daily lives to be able to endure and distract themselves from the pain. They try to find comfortable positions to alleviate the pain. They also avoid movements: *“It feels a little better if I don’t move the legs when I sit still; it’s when I move that it hurts.”* Another way is to lie down to alleviate and endure the pain: *“I feel no pain when I sleep.”* The avoidance of activities and movements in fear of increased level of pain leads to a sedentary lifestyle.

One way to endure and distract from pain is also to consciously transfer the focus from oneself and the pain to things such as participating in associations or watching television: *“I don’t think about the pain when I’m occupied.”* Characteristics such as being resilient, persistent, patient and stubborn or having the ability to *“try to forget about the pain”* are described as successful ways to endure and distract from pain. It gives a sense of being strong when they are able to function despite difficulties: *“You have to confront yourself with the pain to be able to feel that you can pull through despite the pain. It hurts terribly, but I can withstand. In a way, that gives me some strength.”*

The older adults try to take as little medication as possible: *“You don’t want to use unnecessary medication so one tries to manage without it.”* However, it is not possible for the older adults to endure the pain around the clock without medication since there are times when the pain is especially difficult. The endurance and distraction from pain includes balancing the use of medication to relieve pain with the potential side effects such as dizziness, constipation, effects on cognition and risks of falling. *“I’ve gotten worse since I started to take morphine for my back pain. I’ve got difficulties to concentrate and got problems with my stomach, it’s difficult.”*

To endure and distract oneself from pain also requires balancing physical activity without overstraining; *“I feel that I can’t do much in particular…If I do, I’m out.”* It also incorporates balancing emotions and one’s desire *“to be strong and at the same time to allow oneself to be sad.”*

The older adults’ ways to endure and distract from pain to alleviate suffering encompass positive thinking and balancing of activities. This includes such as to rest and avoid movements but also to find ways to keep occupied to hinder thinking of pain.

### Not being taken seriously

Life with pain includes not being taken seriously in times when expressing how bad the pain is or when worrying about cause of pain. Older adults describe the prevailing assumptions that they are old, have lived their lives and that their pain and its underlying cause is not acknowledged the same way as it would be if they were younger, *“…they don’t pay much attention when one becomes old, it doesn’t seem so anyhow.”* The sense of not being taken seriously often undergirds the older adults’ thoughts of being neglected by health care providers. *“…it thrusts and aches and is worst at night. I call the nurse and tell her that it’s bad and that the tears are coming. I tell her that I cry but she says: ‘Just wipe them away’.”* The older adults search for explanations about the cause of their pain. There are worries and fears that something might have gone wrong during procedures as surgery, or that it might be caused by a serious disease like cancer. One woman was finally diagnosed with cancer in her spine after a year of worries and tremendous pain. She was told by the physician that she had nothing to fear and that her problem was related to muscles, but she had strong doubts regarding that explanation. *“When the pain was almost unbearable I thought like this ‘Can muscles really hurt so badly?’ I found it strange that I was treated that way, but maybe I’m a whining biddy? I have always been worried about my skeleton.”*

The older adults’ experience a need to be taken seriously when expressing their worries and fears related to pain. The feeling of not being taken seriously undergirds the sense of being forced to suffer in silence.

### Fear of the future

Life with pain includes fear of the future, but the older adults tend to avoid thinking about the future. There is fear of being forced to a sedentary lifestyle and due to pain, not being able to participate in pleasant activities *“I don’t think about that, I’ll take the sorrow the day it comes, I take the day as it comes.”* There is fear that the situation will become worse as becoming dependent of others and bound to bed, but also a hope that the pain might be gone when one wakes up the next morning. The hesitance to think about the future is related to the experience that: *“It never gets any better, it’s difficult…it makes me low in mood.”* Other thoughts related to the future include: *“The future, I have that behind me, I take it one day at a time, I have nothing…the time is limited.”* There is a resignation of not being able to control the future: *“You never know; you have no control over anything.”* The older adults fear and avoid thinking about the future since they find it uncertain and beyond their control, which might add to the sense of loneliness and suffering in silence.

### Valuing joy and meaning in life

Despite life with pain, the older adults are grateful for the long life they have lived. There is pride in having worked hard in life and reached a high age. They think about things that give joy and meaning in life. The older adults value being able to *“have the strength to get up in the morning, make breakfast, and read.”* They also value their interests and to be occupied with activities such as knitting, cross-word puzzles, or to just sit and look out the window. To listen to a sermon is important for those who have a faith in God.

The older adults’ experiences of dealing with difficulties and losses make it easier to value what gives joy and meaning in life. It also helps them to look at their own suffering in a broader context. They value what they can do instead of focusing on difficulties: *“You have to do the best you can with what you have.”* The older adults value and are proud of their children, grandchildren and great grandchildren since they bring joy and meaning into their lives. This is something that lessens their sense of loneliness and suffering in silence. They value remaining at home with help and support from relatives and health care providers. Having someone who seriously listens helps the older adult to focus on joyful and valuable things in the situation, with significant impact on the sense of well-being and meaning in life.

## Discussion

The findings show that the older adults’ life with long-term musculoskeletal pain is characterized as suffering in silence. The physical, psychological and social restrictions in the older adults’ lives, caused by long-term musculoskeletal pain were obvious in the findings. It also was clear that a life with pain influenced existential values such as integrity, dignity, and independence. In fact, pain influenced the older adults’ access to the world and life itself. The limited access to the world can be understood in light of philosopher Merleau-Ponty (2002), who described the human being as a lived body that is biological and existential at the same time.

The findings, especially the constituents Loneliness and restrictions in daily living and Ways to endure and distract from pain, contributed to the older adult’s sedentary lifestyle and loneliness. These findings can be compared with research by Smith et al. (2019) showing that musculoskeletal pain is associated with increased risk of being lonely but a decreased risk of social isolation in life. The findings in research by Smith and colleagues contrast the findings in this current study and earlier research. The sense of loneliness and lowered mood that were expressed by the older adults in this current study align with earlier research stating an increased risk of depression and social isolation associated with pain (Calvó-Perxas et al. 2016; Gran et al. 2010; Hermsen et al. 2014; Skuladottir and Halldorsdottir 2011). The sedentary lifestyle with limited social interaction and access to the world might worsen the older adults’ situation. The findings in this current study regarding resigning and incorporating the pain as a natural component in the process of aging, is similar to findings by Gillsjö et al. (2012, 2013). However, the findings in Gillsjö et al. (2012) included the components ignoring and struggling as ways to deal with pain in life, which were not explicit in this current study. The finding in this current study also showed that the older adults focused on things that gave joy and meaning in life which also were predominant in research by Gillsjö et al. (2012, 2013) and need to be taken into account in the provision of care.

Unfortunately, the older adults had a sense of not being taken seriously in their effort to express their pain and in searching for answers about its cause. This neglect of their pain and worries might be related to a prevailing opinion among older adults and health care providers that pain is a natural part of aging (c.f. Collis and Waterfield 2015). It is known that this type of pain often is unrecognized, underreported and undertreated (Brown et al. 2011; van der Leeuw et al. 2018) and that older adults tend to have a stoic attitude (Cagle and Bunting 2017; Yong 2006). These factors might add to the fact that the older adults in this study suffered in silence (c.f. Cairncross et al. 2007; Gran et al. 2010).

The suffering caused by pain and not being taken seriously and the fear of the future can be viewed in light of Eriksson’s (1997; 2002) theoretical framework with the three aspects of suffering from illness, care, or life. Suffering from care has been described in relation to experiences of being neglected (Arman et al. 2004), similar to the older adults’ experiences in this study. Research has shown a tendency among nurses to ignore expressions of pain in home health care (Hafskjold et al. 2018). Lack of knowledge and understanding of the older adults’ lifeworld can cause unnecessary suffering that increase their feelings of insecurity, loneliness and alienation (Svanström et al. 2013). The older adults’ suffering in this study contained all three aspects of suffering since it was related to the pain, life itself and the provision of health care.

The findings in this study and earlier research show that the older adults’ needs in relation to pain are not fully acknowledged in the provision of home health care (Gillsjö et al. 2013). This is unfortunate, especially since long-term musculoskeletal pain is a prevailing health problem among older adults and therefor a major reason for consultations in health care. Makris et al. (2014) have highlighted the need of enhanced communication between older adults and their health care providers to improve mutual understanding regarding individual strategies for pain management. In addition, Blyth et al. (2019) have highlighted the need to address the gap in the understanding of the global burden in relation to disabilities caused by musculoskeletal pain conditions. The authors also highlighted the discrepancy between appropriate health care and social care policies and the global burden of musculoskeletal pain conditions. The need of an integrated plan for research and health care is raised as well as the need of a holistic approach to support older adults in finding ways to live their lives with health problems such as pain (Blyth et al. 2019; Kim 2015; Todres et al. 2014).

### Methodological considerations

There was a predominance of women (14 women, 6 men) among the participants which can be viewed as a limitation. However, this type of pain is known to be more prevalent in one or multiple locations, and experienced as disabling to a greater extent by women than by men (Cimas et al. 2018; Jackson et al. 2016; Patel et al. 2013; Rottenberg et al. 2015). Another potential limitation in this study might be that the years living with pain are not described. None of the participants had had pain only 6 months as required for inclusion in the study; instead, they had lived with pain for many years, even decades. The older adults that agreed to participate were willing to be interviewed. There is a risk that older adults with deep sense of loneliness and social isolation did not agree to participate which may influence the findings. A methodological consideration is trustworthiness which in Dahlberg et al. (2008), the originator of RLR, is discussed in terms of validity and objectivity. The validity of the study was further enhanced by the use of qualitative interviews. The participants’ verbal and non-verbal expressions were momentarily and continuously confirmed, and new questions were raised in the interview situation which facilitated thorough descriptions of the participants’ experiences (c.f. Dahlberg et al. 2009; Kvale 1983; 1997). The validity was further enhanced by giving participants time to reflect on adding anything before ending the interview. The findings, however, may have been influenced by the older adults’ tendency to be stoic and view pain as a natural part of aging. These aspects might have limited the older adults’ description of the phenomenon. Objectivity and validity in the study were further enhanced by the authors’ initial readings of the text to become familiar with the data and achieve a sense of the whole. The authors (CG, MB) conducted the analysis and delineated the parts using a reflective and bridling approach throughout the whole research process. The third author (KN) was reading and raised critical reflective question to bridle the understanding (c.f. Dahlberg and Dahlberg 2003; Dahlberg et al. 2008). According to the originators of RLR, Dahlberg et al. (2008), the findings on an abstract level have the potential to be generalized and transferred into similar contexts. However, this is determined by the readers’ earlier experiences, knowledge and context.

## Conclusions

The findings address the importance of increased awareness among health care providers regarding finding ways to alleviate older adults’ suffering in silence in their sedentary life with pain. Increased attentiveness, sensitiveness and an overall person-centered and holistic approach is needed in the provision of care to address the sense of loneliness and support the older adults’ ways to endure long-term musculoskeletal pain with a focus on joy and meaning in life.
